# Traceability of the geographical origin of *Siraitia grosvenorii* based on multielement contents coupled with chemometric techniques

**DOI:** 10.1038/s41598-021-00664-1

**Published:** 2021-10-27

**Authors:** Xiao-Ping Huang, Lei Lei, Shun-Xin Lei, Wei-Wei Zhu, Jun Yan

**Affiliations:** 1 Key Laboratory of Guangxi College and University for Food Safety and Pharmaceutical Chemistry, Guangxi Key Laboratory of Chemistry and Engineering of Forest Products, Guangxi Collaborative Innovation Center for Chemistry and Engineering of Forest Products, School of Chemistry and Chemical Engineering, Guangxi University for Nationalities, Nanning, 530006 People’s Republic of China; 2 School of Chemistry and Life Science, Hechi University, Yizhou, 546300 People’s Republic of China; 3 Hengxian Comprehensive Inspection and Testing Center, Hengxian, 530300 People’s Republic of China

**Keywords:** Analytical chemistry, Metals

## Abstract

*Siraitia grosvenorii* (LHG) is widely used as a medicinal and edible material around the world. The objective of this study was to develop an effective method for the authentication of the geographical origin of LHG in its main producing area Guangxi, China, which is identified as Chinese Protected Designation of Origin product, against other producing regions in China. The content of 14 elements (K, Na, Ca, P, Mg, Al, B, Ba, Cu, Fe, Mn, Ni, Zn, and Sr) of 114 LHG samples was determined by inductively coupled plasma optical emission spectrometry. Multivariate analysis was then performed to classify the geographical origin of LHG samples. The contents of multielement display an obvious trend of clustering according to the geographical origin of LHG samples based on radar plot and principal component analysis. Finally, three supervised statistical techniques, including linear discriminant analysis (LDA), *k*-nearest neighbours (k-NN), and support vector machine (SVM), were applied to develop classification models. Finally, 40 unknown LHG samples were used to evaluate the predictive ability of model and discrimination rate of 100%, 97.5% and 100% were obtained for LDA, *k*-NN, and SVM, respectively. This study indicated that it is feasible to attribute unknown LHG samples to its geographical origin based on its multielement content coupled with chemometric techniques.

## Introduction

It is known that the chemical compositions of plant depend on environmental conditions of a specific geographical area, such as temperature, soil, and rainfall. On the one hand, the chemical composition obviously influences the quality of plant, but on the other hand it also can be used as an indicator to identify the geographical origin of plant. In the past decades, the chemical characterization (based on isotope ratios, trace element composition, organic compounds, or their combinations) coupled with chemometric technique has been used to discriminate geographical origin of plants based products^1–3^. Compared to organic composition profile, often represented by chromatographic fingerprint from HPLC^4^ or GC–MS^5^, element composition profile (also called multielement fingerprint) is more suitable for geographical origin discrimination, due to the element composition of plant is mostly related with its growing environment and easy to determine. Thus, element composition analysis is a promising method for the geographical origin discrimination of a plant based product.

So far, graphite furnace atomic absorption spectrometry (GFAAS), flame atomic absorption spectrometry (FAAS), inductively coupled plasma optical emission spectrometry (ICP-OES), inductively couple plasma mass spectrometry (ICP-MS) and high resolution mass spectrometry (HRMS) have been applied to determine the content of trace elements or stable isotope ratio, combined with supervised pattern recognition techniques, such as linear discriminant analysis (LDA), k-nearest neighbours (*k*-NN), support vector machine (SVM) and artificial neural network (ANN), providing a useful tool for the differentiation of the geographical origin of products. From the reported studies in the literature, this strategy has been successfully applied to agricultural products (cereal^6^, wheat^7,8^, buckwheat^9^, rice^10,11^, onion^12,13^, sesame seed^14^, potato^15^, honey^16^, pumpkin seed oil^17^, chili pepper^18^, cocoa bean^19^, seafood (prawn^20^, fish^21^), fruit (date palm^22^, Sechium edule fruit^23^, lemon juice^24^, meat (wild rabbit meat^25^, tea^26^, pork^27^) and wine^28^.

*Siraitia grosvenorii*, also known as Luo Han Guo (LHG), is a special traditional Chinese medicine which is mainly cultivated in Guangxi, China. LHG is a rich source of a lot of health-promoting phytonutrients, such as polysaccharides, flavonoids, as well as a good source of other antioxidants^29–32^. Plenty of researches indicated that LHG is capable of moisturizing lungs and smoothing coughs, reducing blood pressure, and preventing constipation^33^. For long, LHG has been used as medicine, beverage and food material in China. China's Ministry of Health approved the pharmaceutical/food resource status of LHG, and LHG extracts was registered into the list of generally recognized as safe (GRAS) substance by the US Food and Drug Administration (FDA)^34^. In recent years, important pharmacological characteristics, such as anti-cancer and anti-hyperglycemic effects and inhibition of oxidative modification of low-density lipoprotein, have been reported^35^. Moreover, the LHG from Guangxi has been recognized as Chinese Protected Designation of Origin (PDO) product. However, not all LHG products that are sold as Guangxi LHG on the market are really cultivated in Guangxi. With LHG becoming increasingly popular, some other regions outside of Guangxi began to cultivate LHG. Consequently, the fake brand problem occurs, which not only threaten the livelihood of honest producers, but also infringe the rights of consumers. Thus, a simple and rapid method to differentiate the geographical origin of LHG, especially Guangxi LHG, is required. In our previous studies^36,37^, it has been clearly demonstrated that the combination of near-infrared spectroscopy and chemometric techniques can precisely determine the contents of total phenolic, antioxidant properties and total sugar in LHG. Although the elemental composition of LHG has been sdudied^38,39^, to our knowledge there are no studies using multielement composition as information to discriminate the geographical origin of LHG.

The main objectives of this work were the determination of 14 trace element contents of 114 LHG samples collected from four different regions and the use of chemometric techniques to discriminate the geographical origin of LHG. Accordingly, an ICP-OES method has been applied to determine 14 trace element (K, Na, Ca, P, Mg, Al, B, Ba, Cu, Fe, Mn, Ni, Zn, and Sr) contents of the LHG samples cultivated in Guangxi, Jiangxi, Hunan, and Guizhou province in China. Statistical analysis was performed to reveal the distribution of trace element contents between various regions. In order to discriminate the geographical origin of the LHG samples, pattern recognition techniques such as PCA, LDA, *k*-NN, and SVM were applied.

## Results and discussion

### Statistical description

Firstly, outliers were tested by boxplot technique and replaced by the average value of the rest samples to avoid the loss of information. For each box, the median was used as central mark, the edges of the box represent the 25th and 75th percentiles, and the whiskers extend to the most extreme data points which the algorithm considers to be not outliers. In this work, the whisker parameter was set to 3, thus only the very extreme data points were considered as outliers which might be caused by sample contamination. Finally, 14, 12, 3 and 11 data points were tested as outliers for the multi-elemental measurements of samples in Guangxi, Jiangxi, Hunan, and Guizhou provinces, respectively.

After outlier processing, an approximate normal distribution was found for the element contents of each region. The average and relative standard deviation (RSD) of the trace element contents according to their geographical origins are shown in Table 1. From Table 1, it is obvious that K was the most abundant element in all LHG samples, followed by P, Mg, Ca, Fe, Na, and other trace elements. This result is in accordance with the previous reports^38^, which show that LHG is a good source of these elements, especially K.

**Table 1:** Mean and RSD of the trace element contents according to their origin.

	Guangxi (*N* = 30)		Jiangxi (*N* = 24)		Hunan (*N* = 30)		Guizhou (*N* = 30)		Total (*N* = 114)	
	Mean (μg g^−1 DW^)	RSD (%)	Mean (μg g^−1 DW^)	RSD (%)	Mean (μg g^−1 DW^)	RSD (%)	Mean (μg g^−1 DW^)	RSD (%)	Mean (μg g^−1 DW^)	RSD (%)
K	12,497.6	10.6	13,122.4	12.5	15,947.3	9.4	14,281.2	12.3	14,006.3	14.5
Na	6.0	28.7	4.5	34.4	7.3	33.2	64.6	35.1	21.5	132.3
Ca	294.1	24.9	401.1	23.8	551.3	23.0	813.6	17.4	521.0	43.8
P	1303.9	13.2	1622.5	14.0	2645.3	19.5	2235.0	15.4	1969.0	32.1
Mg	661.1	13.4	719.7	13.0	909.8	19.6	1152.8	15.5	868.3	27.8
Al	7.1	30.7	6.8	33.1	19.1	51.5	28.0	28.9	15.7	70.8
B	6.7	15.1	8.0	24.5	9.7	20.3	13.1	17.0	9.4	32.4
Ba	1.9	31.4	3.1	54.6	1.4	46.5	0.8	34.1	1.7	69.9
Cu	7.3	21.2	5.9	20.5	5.9	20.9	8.0	15.3	6.8	23.8
Fe	31.5	11.5	28.3	21.7	35.4	33.8	63.9	22.0	40.4	43.2
Mn	9.2	27.6	8.7	27.0	6.5	22.1	6.7	37.7	7.7	32.5
Ni	1.3	24.3	1.9	38.7	1.3	36.7	2.0	52.8	1.6	47.8
Zn	11.6	14.6	12.3	14.0	12.9	17.1	22.2	22.5	14.9	36.0
Sr	0.5	23.8	2.1	74.1	0.9	53.5	2.7	24.9	1.5	78.8

In addition, it can be observed that the variations of element content of all samples were much higher than those of individual origin. For example, the RSD of Mg of all samples was 27.8%, which is much larger than the RSD of Mg of an individual origin (13.0–19.6%). However, it should be noted that there were two elements, Mn and Ni, that showing the largest variation (RSD = 37.7% and 52.8% for Mn and Ni, respectively) in Guizhou. We considered that this case was caused by the particular soil characteristics in Guizhou. Generally, large between-groups variability and small within-groups variability is necessary for the good discrimination.

**Figure 1:**
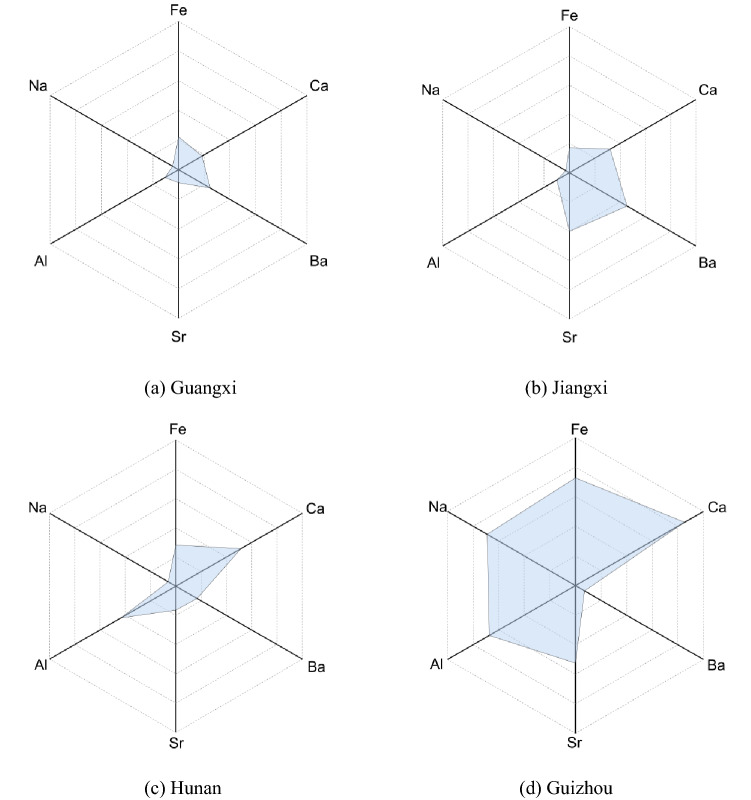
Radar plots showing the difference of geographical origins according to contents of elements (Na, Al, Sr, Ba, Ca, and Fe) in various LHG samples.

Radar plot allows simple, rapid and intuitional discrimination of different patterns, and was, thus, applied in LHG samples to classify their geographical origin by using element content in this study. Six elements (Na, Al, Sr, Ba, Ca, and Fe) with high variations were selected and the average content values were used for radar plots analysis. Figure 1 shows that the distributions of the six element contents of LHG samples from different regions showed obviously different characteristic patterns. For instance, all elements except Ba had higher contents in Guizhou than the other three regions. It should also be noted that the LHG samples of Guangxi have the lowest content values for five elements (Na, Al, Sr, Ca, and Fe). Radar plot shows that multielement content has the potential to be used for the discrimination of geographical origin of LHG.

### Principal component analysis

At preliminary stage, PCA was used for exploratory data analysis before classification modeling. PCA is a commonly used dimension reduction technique, which provides the distributions of samples by projecting them on a set of orthogonal basis. In this study, the 74 LHG samples of training set and 14 element contents formed the input data matrix (74 rows and 14 columns) and then was analysed by PCA based on singular value decomposition algorithm.

**Figure 2:**
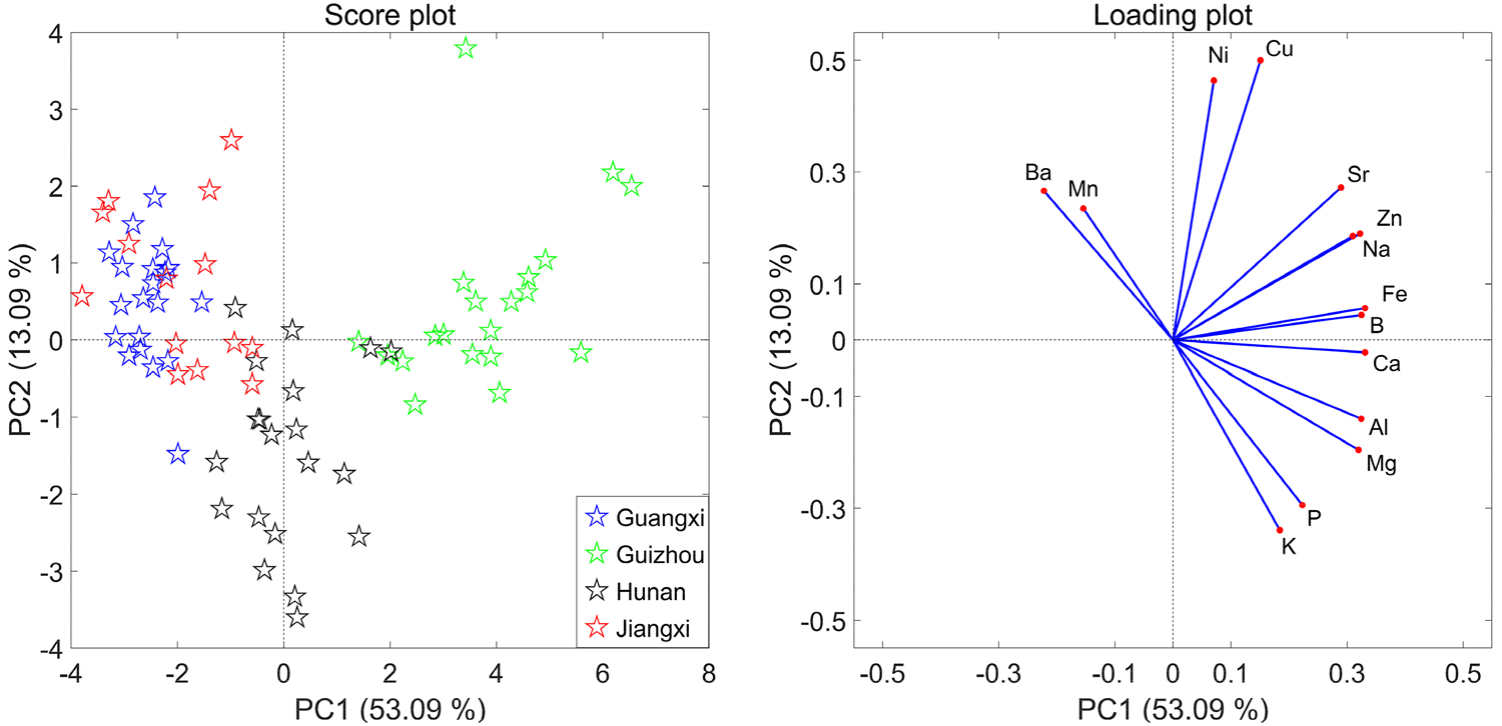
Score and loading plots of PC1 versus PC2.

As shown in Fig. 2, the first principal component (PC1) and the second principal component (PC2) can explain 53.09% and 13.09% of the total variance, respectively. The score plot shows a clear separation pattern for the samples from Guizhou and the other three regions, meanwhile, the samples of Hunan are well separated from those of Guizhou, Jiangxi and Guangxi. This distribution can be interpreted from the loading plot that indicated the content of Fe (63.9 μg g^−1^), B (13.1 μg g^−1^), Ca (813.6 μg g^−1^), Na (64.6 μg g^−1^) and Zn (22.2 μg g^−1^) is higher for samples from Guizhou, thus the samples from Guizhou are obviously separated with others based on PC1. As for samples from Hunan, they are separated with other samples based on PC2, due to the high content of K (15,947.3 μg g^−1^) and P (2645.3 μg g^−1^), and the low content of Ni (1.3 μg g^−1^) and Cu (5.9 μg g^−1^) for these samples. The higher K and P contents may be caused by excessive fertilization in Hunan. However, a serious overlapping between the samples from Guangxi and Jiangxi was observed. From Figs. 1 and 2, it can be seen that there is a farther distance between Jiangxi and Guangxi, but their element profile is more similar, which leads to the overlapping in PCA analysis. In order to obtain reliable classification models for different LHG samples, supervised learning pattern recognition techniques were applied.

### Supervised classification models

In this work, the models for the classification of LHG samples according to their geographical origin were developed using three supervised pattern recognition techniques with different mechanisms, including LDA, *k*-NN, and SVM. LDA is a linear classification technique by maximizing the variance between classes and minimizing the variance within each class. Discriminant functions were constructed by the linear combinations of original variables and used to differentiate groups of samples. The test set is predicted by the projection of the new samples according the minimal distance to the centroid of each class. Compared to PCA, LDA is a supervised method which uses the labels of samples in training set to develop model. Thus, LDA can give a better pattern recognition result than PCA.

**Figure 3:**
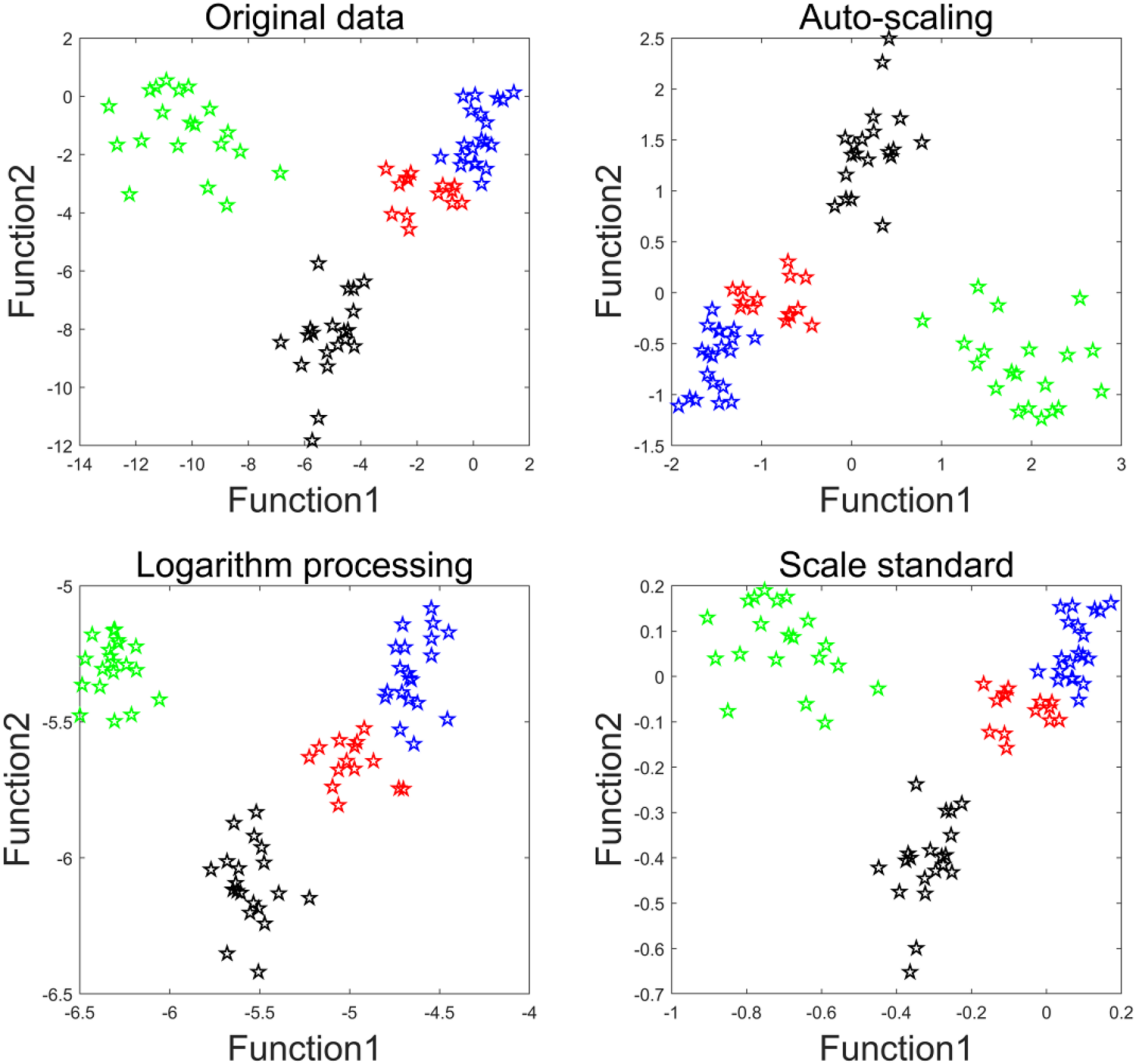
The classification of 95 LHG samples by LDA based on different data pre-processing methods (blue: Guangxi; black: Hunan; green: Guizhou; red: Jiangxi).

At first, the original element content values were treated by three data pre-processing methods, including auto-scaling, scale standard, and logarithm processing. To put it simply, auto-scaling returns the results for each element of the original data set ***X*** such that columns of ***X*** are centered to have mean 0 and scaled to have standard deviation 1, scale standard processes the original data set ***X*** by normalizing the minimum and maximum values of each row, and logarithm processing directly to take the log of the original data. Data pre-processing can eliminate the effect of the order of magnitudes, and included all information of original data. After data pre-processing, the 14 elemental content values were used in LDA analysis and the distribution of 74 samples in training set were shown in Fig. 3. It is observed that all samples were clearly separated based on function 1 and function 2, and logarithm processing give the best classification result. Therefore, logarithm processing was used in this work, and three discriminant functions explained the 100% of the variance (function 1 explained the 77.95% of the total variance; function 2 the 19.42%; function 3 the 2.63%). Function 1 and function 2 are as follows:

1 $$ {\text{F1}} = 0.{\text{2291K }} - \, 0.{\text{2412Na }} - \, 0.{\text{4323Ca }} - \, 0.{\text{5638P }} - \, 0.{\text{2127Mg }} - \, 0.{1}0{\text{95Al}} + 0.0{\text{941B }} + \, 0.{\text{2666Ba }} + \, 0.{\text{1668Cu }} + \, 0.{\text{4321Fe }} - \, 0.0{\text{922Mn }} - \, 0.0{\text{572Ni}} - 0.{\text{1286Zn }} - \, 0.0{\text{682Sr}} $$

2 $$ {\text{F2}} = - 0.{\text{4254K }} + \, 0.{15}0{\text{5Na }} - \, 0.0{\text{419Ca }} - \, 0.{6}0{\text{32P }} + \, 0.{1}0{\text{15Mg }} - \, 0.{\text{2247Al}} - 0.0{\text{473B }} - \, 0.0{\text{518Ba }} + \, 0.{\text{2672Cu }} + \, 0.{49}00{\text{Fe }} + \, 0.{\text{1391Mn }} - \, 0.{1}0{\text{52Ni}} + \, 0.{152}0{\text{Zn }} + \, 0.00{\text{76Sr}} $$

It should be noted that the most effective element for the geographical origin discrimination of LHG samples were those with the highest absolute correlation within discriminant function. In particular, for the function 1 there were P, Ca, and Fe with absolute correlation of 0.5638, 0.4323, and 0.4321, respectively. Similarly, for the function 2 there were P, Fe, and K with absolute correlation of 0.6032, 0.4900, and 0.4254, respectively. Given the fact that function 1 and function 2 accounted for 97.37 of total variance, thus the most effective elements for the geographical origin discrimination of LHG were P, Fe, Ca, and K.

**Figure 4:**
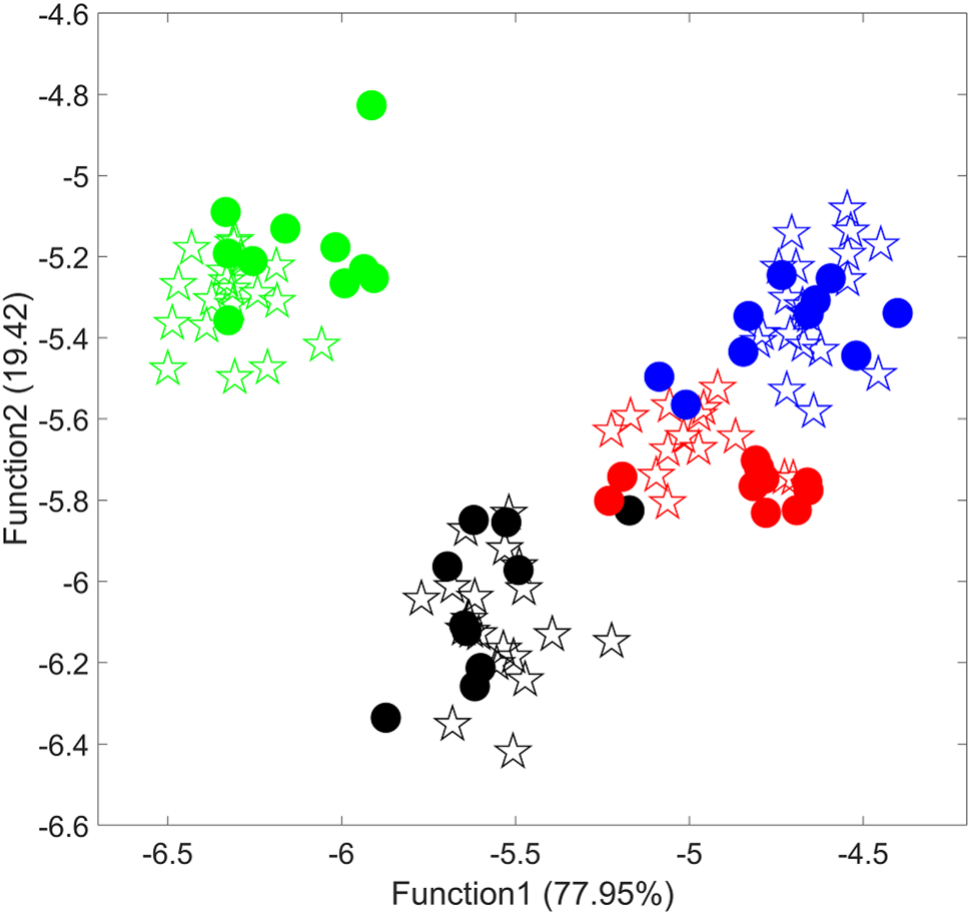
The distribution of samples in training set and test set (☆ training set; ● test set; blue: Guangxi; black: Hunan; green: Guizhou; red: Jiangxi).

Five-fold cross validation was used to evaluate the classification ability of LDA for the 74 samples in training set. As shown in Table 3, the accuracy for training set is 100%, and all samples were correctly grouped according to their geographical origin. For the test set, 40 unknown samples were used to evaluate the predictive ability of the classifier based on LDA. The samples were projected based on function 1 and function 2. As shown in Fig. 4, it is obvious that all the samples in test set were almost properly classified. Although there is a slight overlapping between the samples from Guangxi and Jiangxi, function 3 can further improve the classification effect. Finally, all samples of test set were correctly classified by LDA, the result are shown in Table 2. In addition, LDA based on other data pre-processing methods also provides acceptable classification ability, with accuracy range from 95% to 97.5%. The result of origin data without any pre-processing is the worst (90%). Details can be found in Table S2 in Supplementary material.

**Table 2:** The discrimination results of different models for the train set and the test set.

Geographical origins	Number of samples		LDA accuracy (%)		*k*-NN accuracy (%)		SVM accuracy (%)	
	Training set	Test set	Training set	Test set	Training set	Test set	Training set	Test set
Guangxi	20	10	100	100	95	100	100	100
Jiangxi	14	10	100	100	93	100	100	100
Hunan	20	10	100	100	100	100	95	100
Guizhou	20	10	100	100	100	90	100	100
Total accuracy (%)			100	100	97	97.5	98.7	100

Besides LDA, *k*-NN and SVM were used to develop classification models, and the results of classification are shown in Table 2 (The accuracy for training set is calculated using fivefold cross validation). *k*-NN analysis is a distance based technique for pattern recognition, which is easy to understand and implement. In short, this technique assigns an unknown sample into the class most common among its *k*-nearest neighbours according to distance. In this work, Euclidean distance was used and the optimal size of neighbours *k* (*k* = 3) was optimized using five-fold cross validation procedure by which maximum accuracy rate was selected as criterion. SVM uses a nonlinear mapping to transform the original training data into a higher dimension, and then finds a hyperplane using support vectors and margins to classify the data. Through the above analysis, we observed that the three models showed different degrees of success, and LDA and SVM performed better than *k*-NN. For the three methods, the classification accuracy for training set and test set was 97–100% and 97.5–100%, respectively. These results demonstrate that the element content is an effective approach for the classification of LHG samples according to geographical origin.

The main objective of the study described here was to apply ICP-OES analysis, combined with chemometric techniques, to develop a method for discriminating the geographical origin of LHG samples. Statistical analysis showed that there was an obvious impact of geographical origin on element content values in LHG, especially for P, Fe, Ca, and K. Three classification models based on LDA, *k*-NN and SVM in the present work suggesting the great potential of element content for the geographical traceability of LHG. Furthermore, in order to further improve the robustness and accuracy of classification model, more LHG samples should be analysed in the future from different harvest years.

## Materials and methods

### Reagents and apparatus

Nitric acid (HNO_3_, 65% AR) used for sample preparation were purchased from Aladdin (Shanghai, China). Stock solutions of a multi-elemental standard solution (Serial No.: GSB04-1767-2004, 100 μg mL^−1^) containing eight elements (Al, Mg, B, Cu, Mn, Ni, Zn, and Sr) from Guobiao (Beijing) Testing and Certification Co., Ltd. (Beijing, China), and six single element standard solution of Ca (GSB G 62012-90(2001), 1000 μg mL^−1^), P (GSB G 62008-90(1503), 1000 μg mL^−1^), K (GBW(E)080259, 1000 μg mL^−1^), Na (GSB G 62004-90(1101), 1000 μg mL^−1^), Ba(GSBG62046-90(5601), 1000 μg mL^−1^), and Fe(GSBG62020-90(2601), 1000 μg mL^−1^) from National Analysis Centre for Iron & Steel (Beijing, China) were used for element content calibration. A standard reference material (onion sample, CAS: GBW10049-GSB-27) from the Institute of Geology and Geochemistry (Hebei, China), was employed in order to assure the accuracy of the whole procedure. Deionised water was obtained from a Milli-Q system (Millipore, Bedford, MA, USA).

A high throughput closed microwave digestion system, CEM Mars6 from CEM (USA), was used for the microwave-assisted digestion of LHG samples. An electrothermal digestion apparatus EHD-24 from Oriental Innovation Biotechnology Co., Ltd (Beijing, China), was applied to eliminate the nitrous vapours after microwave digestion. Multi-elemental trace analysis of previously digested samples was carried out using an inductively coupled plasma optical emission spectrometer (Prodigy, Teledyne Leeman Labs, USA).

The plastic containers used for treating and storing LHG samples were cleaned by overnight immersion in nitric acid solution (2%, v/v), rinsed with ultrapure water and dried, to avoid contamination of samples with traces of any other metal.

### Sample pre-treatment

A total of 114 LHG samples (All samples were processed by drying in an oven) were collected in 2019 from local producers of four producing regions in China, including Guangxi (East longitude 104°26′-112°04′, North latitude 20° 54′–26° 24′, *N* = 30), Jiangxi (East longitude 113° 34′–118° 28′, North latitude 24° 29′–30° 04′, *N* = 24), Hunan (East longitude 108° 47′–114° 15′, North latitude 24° 38′–30° 08′, *N* = 30), and Guizhou (East longitude 103° 36′–109° 35′, North latitude 24° 37′–29° 13′, *N* = 30) provinces. These samples have been done the formal identification by Lei Lei from Hengxian Comprehensive Inspection and Testing Center. Next, these samples were ground by a DFY-200C Mill (Shanghai Sirui Instruments Co.LTD, China) and the obtained powder was sieved through a 0.42 mm mesh screen. The large particles which cannot pass through the mesh were ground again. All the methods used in the study for the plant materials are in compliance with the guidelines and legislation of China.

Powder samples (1 g) of LHG were weighed inside Teflon digestion vessels, and 8 mL of concentrated nitric acid was added quickly. After the vessels were capped, they were placed in the microwave oven and the following program was run: step1, 10 min to reach 120 ℃ and keep 15 min; step 2, 5 min to reach 150 ℃ and keep 10 min; step 3, 10 min to reach190 ℃ and keep 40 min; step 4, 15 min to reach 180 ℃ and keep 15; step 5, 15 min to reach 100 ℃ and keep 10 min, and step 6, cooling down to room temperature. Next, the vessels were opened and the droplets on the inner cap were rinsed into vessels by distilled water. Then, the vessels were placed on the electrothermal digestion apparatus at 120 ℃ to eliminate the nitrous vapours. Finally, the sample was transferred into a 25 mL volumetric flask for future use.

### Elements measurement

The content of 14 elements (K, Na, Ca, P, Mg, Al, B, Ba, Cu, Fe, Mn, Ni, Zn, and Sr) was measured using an ICP-OES (Prodigy, Teledyne Leeman Labs, USA). The power was set to 1100 W and the selected analytical emission lines (nm) were automatically determined by the instrument. The calibration standards were prepared from a multi-elemental standard solution and six single standard solutions. Calibration curves were obtained at five different content levels in triplicate, and calibration ranges were properly set according to the expected mineral content range in LHG samples. The accuracy of the measurement was verified by analyzing a standard reference material (onion sample, CAS: GBW10049-GSB-27). The standard reference material and the LHG samples were analysed under the same conditions. Each sample was digested and analysed three times and the average contents were used in the subsequent data analysis. The information of analytical conditions and calibration parameters are summarized in Table 3.

**Table 3:** Summary of calibration parameters and analytical conditions.

	Analysis line (nm)	Calibration range (mg/L)	R^2^
K	766.49	1–50	0.9998
Na	589.59	0.1–10	0.9999
Ca	422.67	0.1–20	0.9999
P	253.56	1–50	0.9999
Mg	279.07	0.5–50	0.9999
Al	394.40	0.2–5	0.9999
B	208.95	0.02–1	0.9999
Ba	455.40	0.02–1	0.9999
Cu	324.75	0.02–1	0.9999
Fe	238.20	0.02–5	0.9999
Mn	257.61	0.02–1	0.9999
Ni	231.60	0.02–1	0.9999
Zn	213.85	0.02–1	0.9999
Sr	407.77	0.02–1	0.9999

### Statistical analysis and chemometrics

Several statistical analysis methods and chemometric techniques were used to visualize the data structure and to classify the LHG samples according to their geographical origin. At first, the data matrix was tested by applying boxplot technique for outlier detection. After outlier processing, some important statistical parameters were calculated and represented by radar plot. Next, PCA was used to display the distribution of LHG samples based on the first two principal components. Finally, three supervised pattern recognition techniques, i.e. LDA, *k*-NN, and SVM, were applied to develop pattern recognition models.

For the three supervised recognition techniques, namely LDA, *k*-NN, and SVM, 114 LHG samples were randomly divided into a training set (*N* = 74) and a test set (*N* = 40). In order to balance the class distributions within the divisions, the uniform sampling was carried out according to the geographical origin of samples. Finally, a training set consisting of 74 samples (20, 14, 20, and 20 samples in Guangxi, Jiangxi, Hunan, and Guizhou, respectively.) and a test set consisting of 40 samples (10, 10, 10, and 10 samples in Guangxi, Jiangxi, Hunan, and Guizhou, respectively.) were obtained (Details can be found in Table S1 in Supplementary material). A training set with known class labels was used to calculate a classifier and a test set was used to evaluate the performance of the classifier.

*k*-fold cross validation is commonly used to assess the classification ability of the model and to optimize the parameters of algorithms. For *k*-fold cross validation, samples are divided into *k* mutuality exclusive subsets of similar size, the prediction capacity of model is assessed by the average result of *k* runs in cross validation. In this work, five-fold cross validation was applied. The software Matlab R2015a (MathWorks, USA) was used for all calculations and programming on a personal computer.

## Supplementary Information

Supplementary Information.
