# Supplementary material for: Traceability of the geographical origin of Siraitia grosvenorii based on multielement contents coupled with chemometric techniques
Source: Sci Rep. 2021 Oct 27;11:21150. doi: 10.1038/s41598-021-00664-1 (PMC8551321; doi:10.1038/s41598-021-00664-1)
Supplement: Supplementary file 1 — Supplementary Information. [file 41598_2021_664_MOESM1_ESM.docx]

**Supplementary material**


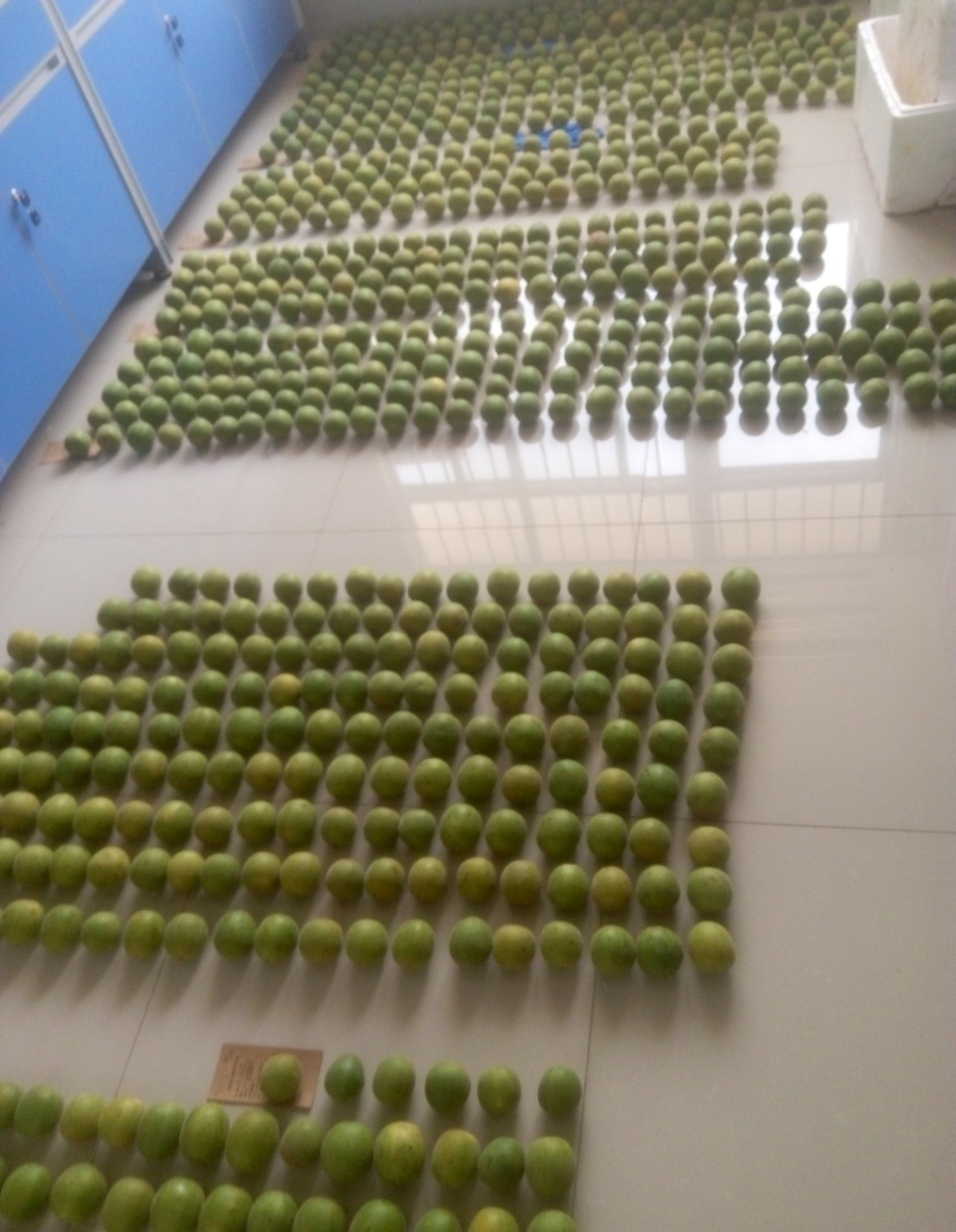

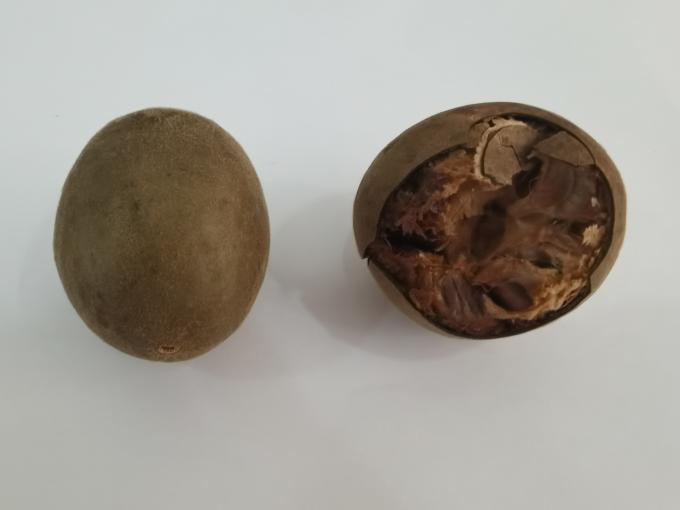


Figure S1 pictures of Siraitia grosvenorii in laboratory (Left: fresh fruit, Right: dried fruit), Photographed by Jun Yan.

Table S1 Element content values of samples in training set and test set

| **Training set (N=74)** | | | | | | | | | | | | | | |
| --- | --- | --- | --- | --- | --- | --- | --- | --- | --- | --- | --- | --- | --- | --- |
|  | K | Na | Ca | P | Mg | Al | B | Ba | Cu | Fe | Mn | Ni | Zn | Sr |
| **1** | 11980 | 8.22 | 309 | 1069 | 540 | 7.13 | 7.32 | 1.66 | 7.00 | 40.38 | 8.43 | 1.18 | 10.68 | 0.56 |
| **2** | 11269 | 7.78 | 230 | 1189 | 617 | 5.67 | 7.01 | 2.23 | 8.27 | 29.79 | 9.86 | 1.26 | 12.02 | 0.49 |
| **3** | 11427 | 7.78 | 305 | 1118 | 663 | 6.41 | 6.72 | 1.77 | 5.99 | 26.43 | 6.71 | 0.70 | 9.10 | 0.52 |
| **4** | 14979 | 8.35 | 251 | 1204 | 685 | 5.23 | 7.89 | 1.56 | 6.31 | 27.66 | 8.55 | 0.95 | 9.86 | 0.52 |
| **5** | 0 | 4.39 | 299 | 1442 | 655 | 7.52 | 6.60 | 2.23 | 5.56 | 31.56 | 9.22 | 1.12 | 10.52 | 0.54 |
| **6** | 11683 | 9.68 | 276 | 1154 | 659 | 6.54 | 8.66 | 2.76 | 5.59 | 24.47 | 8.86 | 1.11 | 9.52 | 0.57 |
| **7** | 12999 | 6.12 | 246 | 1418 | 720 | 7.37 | 8.66 | 2.07 | 7.81 | 28.50 | 10.03 | 1.25 | 12.67 | 0.49 |
| **8** | 10687 | 6.38 | 324 | 1307 | 574 | 4.91 | 5.98 | 2.94 | 6.93 | 26.31 | 8.86 | 1.17 | 11.34 | 0.50 |
| **9** | 15615 | 5.66 | 452 | 1348 | 803 | 7.06 | 8.56 | 2.16 | 6.93 | 37.53 | 10.81 | 1.51 | 12.59 | 0.64 |
| **10** | 12302 | 7.77 | 314 | 1152 | 578 | 7.16 | 8.02 | 1.57 | 7.81 | 33.02 | 8.98 | 1.62 | 11.50 | 0.51 |
| **11** | 12341 | 1.85 | 321 | 1440 | 722 | 5.12 | 5.59 | 1.69 | 8.24 | 31.18 | 8.67 | 1.10 | 8.60 | 0.48 |
| **12** | 11427 | 6.40 | 326 | 1182 | 637 | 6.13 | 6.59 | 1.29 | 8.74 | 29.88 | 7.35 | 1.02 | 15.08 | 0.17 |
| **13** | 13273 | 6.25 | 267 | 1599 | 765 | 5.26 | 5.82 | 1.53 | 8.87 | 33.27 | 8.93 | 1.15 | 8.98 | 0.22 |
| **14** | 11683 | 4.29 | 446 | 1305 | 848 | 8.65 | 5.60 | 3.21 | 7.97 | 37.28 | 13.84 | 1.26 | 13.77 | 0.70 |
| **15** | 11010 | 4.01 | 275 | 1294 | 680 | 4.20 | 4.97 | 2.23 | 7.45 | 34.39 | 9.15 | 1.14 | 10.43 | 0.48 |
| **16** | 11476 | 4.07 | 381 | 1161 | 629 | 12.81 | 6.17 | 1.95 | 5.02 | 34.16 | 8.73 | 1.21 | 9.56 | 0.63 |
| **17** | 12094 | 6.84 | 220 | 1167 | 697 | 8.47 | 6.61 | 2.26 | 6.73 | 32.73 | 8.80 | 1.43 | 14.70 | 0.48 |
| **18** | 10714 | 8.01 | 152 | 973 | 510 | 6.81 | 5.69 | 1.08 | 5.17 | 27.71 | 6.70 | 1.06 | 10.83 | 0.85 |
| **19** | 14379 | 4.04 | 258 | 1228 | 860 | 5.64 | 7.29 | 1.11 | 4.35 | 34.58 | 5.81 | 0.76 | 10.43 | 0.80 |
| **20** | 11915 | 7.21 | 306 | 1026 | 695 | 8.69 | 7.38 | 1.28 | 4.53 | 31.09 | 4.97 | 1.67 | 12.70 | 0.48 |
| **21** | 18483 | 115.46 | 866 | 2252 | 1265 | 16.90 | 15.02 | 0.68 | 8.86 | 56.61 | 7.03 | 2.09 | 23.89 | 2.41 |
| **22** | 15268 | 95.43 | 881 | 1858 | 949 | 21.33 | 16.28 | 0.56 | 6.86 | 49.66 | 3.66 | 1.11 | 21.62 | 2.60 |
| **23** | 13022 | 68.84 | 824 | 2719 | 1244 | 48.47 | 17.13 | 1.09 | 9.12 | 92.57 | 9.32 | 2.63 | 28.34 | 2.43 |
| **24** | 11883 | 72.52 | 973 | 1938 | 877 | 23.70 | 13.38 | 0.67 | 7.61 | 53.21 | 4.02 | 0.81 | 19.37 | 3.55 |
| **25** | 18331 | 79.79 | 1019 | 2522 | 1201 | 36.85 | 16.11 | 0.79 | 9.53 | 69.59 | 8.08 | 2.85 | 27.92 | 3.85 |
| **26** | 13405 | 42.81 | 797 | 1805 | 1038 | 21.73 | 12.69 | 0.67 | 5.95 | 45.06 | 5.96 | 0.90 | 19.10 | 2.60 |
| **27** | 11845 | 60.92 | 773 | 2371 | 1000 | 34.01 | 12.92 | 0.67 | 7.65 | 62.43 | 5.00 | 1.23 | 22.30 | 2.45 |
| **28** | 14452 | 57.18 | 698 | 2054 | 1020 | 21.84 | 13.71 | 0.52 | 7.43 | 49.15 | 5.35 | 1.12 | 20.37 | 2.34 |
| **29** | 15153 | 71.34 | 920 | 2055 | 1195 | 27.14 | 12.89 | 0.78 | 7.82 | 62.74 | 5.70 | 1.26 | 19.98 | 3.58 |
| **30** | 17845 | 35.82 | 884 | 2433 | 1214 | 29.98 | 14.22 | 0.82 | 9.27 | 57.83 | 7.41 | 1.79 | 25.10 | 2.80 |
| **31** | 13115 | 88.14 | 895 | 1834 | 990 | 22.98 | 10.39 | 0.52 | 11.57 | 52.59 | 3.63 | 4.76 | 14.38 | 2.44 |
| **32** | 13089 | 31.03 | 624 | 1963 | 1038 | 26.03 | 8.61 | 0.63 | 6.59 | 50.64 | 6.10 | 1.78 | 16.31 | 1.78 |
| **33** | 12586 | 55.86 | 692 | 2862 | 1367 | 29.79 | 9.99 | 0.71 | 7.71 | 62.19 | 5.11 | 1.26 | 20.33 | 2.39 |
| **34** | 15016 | 52.44 | 689 | 2363 | 1137 | 23.25 | 11.48 | 0.49 | 7.15 | 47.08 | 4.23 | 1.23 | 17.29 | 1.59 |
| **35** | 14622 | 103.49 | 863 | 1905 | 1209 | 29.46 | 12.09 | 0.67 | 7.26 | 59.67 | 4.35 | 0.80 | 16.28 | 2.87 |
| **36** | 14224 | 47.85 | 1121 | 2449 | 1351 | 29.09 | 11.56 | 0.74 | 5.98 | 58.23 | 5.38 | 1.22 | 18.76 | 3.22 |
| **37** | 15103 | 108.94 | 787 | 2698 | 1552 | 38.14 | 13.20 | 1.11 | 7.89 | 72.59 | 4.67 | 1.17 | 22.08 | 2.63 |
| **38** | 12740 | 59.89 | 498 | 2524 | 1114 | 21.00 | 10.67 | 0.39 | 7.90 | 47.52 | 5.41 | 1.21 | 19.26 | 1.05 |
| **39** | 14167 | 79.32 | 988 | 2467 | 1342 | 42.78 | 12.61 | 1.05 | 7.03 | 74.12 | 12.17 | 1.77 | 19.21 | 2.87 |
| **40** | 15190 | 78.26 | 859 | 2002 | 1150 | 27.09 | 10.26 | 1.25 | 6.22 | 61.89 | 8.38 | 1.15 | 17.42 | 2.81 |
| **41** | 16918 | 6.37 | 360 | 3318 | 779 | 9.82 | 10.02 | 1.39 | 7.96 | 31.05 | 5.44 | 1.84 | 16.49 | 0.76 |
| **42** | 14842 | 8.33 | 572 | 2777 | 896 | 18.30 | 8.46 | 2.22 | 7.08 | 32.58 | 6.14 | 2.32 | 14.14 | 1.45 |
| **43** | 16033 | 8.01 | 539 | 3506 | 756 | 12.67 | 8.76 | 2.08 | 6.56 | 26.35 | 6.62 | 1.70 | 11.18 | 1.00 |
| **44** | 14451 | 7.35 | 583 | 2150 | 796 | 8.46 | 8.52 | 2.46 | 6.59 | 23.05 | 6.14 | 2.15 | 12.64 | 1.55 |
| **45** | 16132 | 5.51 | 476 | 2355 | 913 | 25.75 | 8.04 | 0.85 | 3.11 | 40.56 | 6.54 | 0.65 | 9.73 | 0.94 |
| **46** | 15563 | 6.26 | 471 | 2822 | 732 | 8.14 | 10.28 | 1.56 | 6.93 | 22.36 | 6.28 | 2.04 | 13.24 | 1.17 |
| **47** | 15628 | 11.94 | 564 | 3088 | 923 | 31.05 | 11.32 | 1.31 | 8.09 | 45.87 | 5.03 | 2.30 | 18.00 | 1.34 |
| **48** | 15596 | 8.85 | 599 | 3109 | 1040 | 23.62 | 14.12 | 1.58 | 7.66 | 44.99 | 8.62 | 2.14 | 14.82 | 1.19 |
| **49** | 19672 | 5.69 | 482 | 2902 | 1153 | 14.46 | 10.35 | 1.04 | 7.16 | 42.09 | 7.98 | 1.19 | 15.88 | 0.93 |
| **50** | 14588 | 7.09 | 607 | 2270 | 964 | 18.72 | 7.95 | 0.98 | 6.49 | 36.81 | 5.51 | 1.04 | 13.73 | 1.33 |
| **51** | 14247 | 5.72 | 760 | 2342 | 973 | 15.06 | 8.94 | 2.27 | 4.65 | 30.24 | 7.35 | 1.44 | 11.54 | 1.14 |
| **52** | 15592 | 8.47 | 476 | 1784 | 960 | 11.42 | 9.21 | 0.94 | 3.48 | 27.60 | 7.25 | 0.90 | 10.02 | 0.69 |
| **53** | 17701 | 12.09 | 632 | 2178 | 1184 | 19.39 | 8.85 | 2.04 | 5.60 | 42.72 | 7.32 | 1.17 | 12.96 | 0.87 |
| **54** | 15326 | 6.61 | 466 | 2387 | 1066 | 24.05 | 7.83 | 0.59 | 4.47 | 38.66 | 6.28 | 1.06 | 11.82 | 0.19 |
| **55** | 16313 | 4.99 | 679 | 3388 | 927 | 16.17 | 7.50 | 2.15 | 6.00 | 28.14 | 5.71 | 0.90 | 10.42 | 0.13 |
| **56** | 16488 | 4.02 | 522 | 2454 | 1081 | 28.48 | 6.91 | 0.57 | 3.59 | 44.03 | 5.13 | 0.85 | 12.87 | 0.10 |
| **57** | 17012 | 5.08 | 624 | 3006 | 1249 | 29.58 | 9.85 | 0.91 | 5.28 | 50.48 | 7.59 | 1.16 | 13.81 | 0.37 |
| **58** | 13811 | 2.94 | 476 | 2490 | 734 | 12.26 | 7.57 | 0.63 | 5.55 | 24.29 | 5.46 | 1.28 | 11.71 | 0.19 |
| **59** | 17348 | 7.55 | 550 | 2348 | 1147 | 32.35 | 7.33 | 0.71 | 3.54 | 44.49 | 6.36 | 0.59 | 9.52 | 0.29 |
| **60** | 14564 | 2.95 | 659 | 2445 | 1067 | 21.74 | 8.97 | 2.06 | 6.05 | 38.48 | 7.48 | 1.33 | 10.90 | 0.23 |
| **61** | 11895 | 7.62 | 269 | 2119 | 727 | 7.03 | 3.69 | 1.61 | 9.52 | 27.67 | 5.55 | 2.60 | 13.45 | 1.98 |
| **62** | 14436 | 3.93 | 484 | 1851 | 854 | 8.21 | 8.50 | 2.08 | 5.56 | 30.48 | 11.19 | 0.84 | 11.03 | 1.29 |
| **63** | 11531 | 3.70 | 417 | 1680 | 650 | 9.00 | 6.32 | 1.75 | 5.26 | 32.58 | 7.67 | 1.09 | 13.74 | 1.08 |
| **64** | 12321 | 5.47 | 523 | 1474 | 718 | 9.08 | 7.77 | 5.34 | 3.87 | 30.61 | 13.08 | 1.19 | 11.47 | 1.94 |
| **65** | 14526 | 3.22 | 396 | 1976 | 851 | 10.08 | 10.80 | 1.40 | 5.65 | 34.54 | 7.79 | 1.21 | 16.27 | 0.97 |
| **66** | 16343 | 3.59 | 425 | 1662 | 753 | 8.17 | 7.51 | 4.50 | 7.43 | 37.38 | 7.69 | 3.42 | 15.52 | 2.35 |
| **67** | 12405 | 4.07 | 309 | 1370 | 692 | 3.21 | 7.89 | 1.40 | 5.80 | 20.10 | 8.06 | 2.08 | 12.09 | 1.53 |
| **68** | 12333 | 4.41 | 456 | 1526 | 632 | 6.13 | 7.16 | 1.34 | 5.58 | 27.41 | 6.53 | 0.81 | 13.15 | 0.93 |
| **69** | 13026 | 5.16 | 525 | 1740 | 754 | 8.70 | 10.20 | 1.53 | 5.49 | 37.28 | 8.31 | 1.35 | 14.60 | 1.08 |
| **70** | 12734 | 2.57 | 293 | 1460 | 653 | 5.25 | 7.06 | 3.08 | 5.43 | 21.52 | 15.91 | 2.10 | 11.27 | 1.40 |
| **71** | 14480 | 7.42 | 311 | 1609 | 825 | 4.10 | 7.52 | 1.58 | 6.98 | 23.26 | 5.33 | 3.17 | 11.69 | 1.08 |
| **72** | 15819 | 4.33 | 630 | 1498 | 691 | 11.70 | 10.52 | 1.49 | 7.22 | 40.59 | 8.50 | 1.06 | 11.31 | 1.19 |
| **73** | 9425 | 7.63 | 268 | 1527 | 643 | 4.33 | 5.85 | 2.85 | 6.14 | 21.07 | 8.45 | 2.43 | 10.26 | 0.53 |
| **74** | 10961 | 7.16 | 253 | 1520 | 526 | 3.17 | 4.66 | 2.99 | 5.15 | 17.51 | 7.37 | 1.49 | 9.75 | 0.84 |
| **Test set (N=40)** | | | | | | | | | | | | | | |
|  | K | Na | Ca | P | Mg | Al | B | Ba | Cu | Fe | Mn | Ni | Zn | Sr |
| **1** | 13590 | 5.10 | 177 | 1395 | 572 | 3.81 | 6.22 | 1.59 | 6.68 | 30.60 | 7.13 | 1.09 | 11.59 | 0.57 |
| **2** | 12892 | 5.66 | 264 | 1336 | 601 | 5.58 | 6.08 | 0.80 | 7.27 | 29.48 | 7.15 | 1.22 | 11.28 | 0.57 |
| **3** | 12366 | 4.27 | 256 | 1200 | 539 | 6.22 | 5.27 | 0.83 | 7.26 | 32.01 | 6.54 | 1.13 | 10.76 | 0.57 |
| **4** | 13593 | 6.44 | 341 | 1641 | 736 | 11.09 | 6.05 | 2.07 | 9.47 | 28.65 | 11.39 | 2.02 | 14.15 | 0.57 |
| **5** | 14853 | 8.27 | 455 | 1507 | 770 | 11.03 | 7.62 | 2.44 | 7.89 | 28.65 | 16.93 | 1.20 | 12.61 | 0.52 |
| **6** | 12345 | 5.47 | 330 | 1391 | 627 | 11.59 | 7.03 | 1.41 | 10.82 | 28.65 | 8.52 | 1.20 | 12.11 | 0.57 |
| **7** | 11099 | 5.31 | 316 | 1478 | 634 | 6.06 | 7.10 | 1.95 | 9.37 | 34.24 | 10.97 | 1.91 | 13.64 | 0.57 |
| **8** | 12197 | 4.59 | 232 | 1588 | 617 | 6.91 | 6.09 | 1.77 | 9.07 | 33.98 | 14.19 | 1.86 | 12.79 | 0.57 |
| **9** | 11214 | 5.30 | 298 | 1367 | 581 | 6.30 | 6.08 | 2.40 | 8.34 | 34.30 | 10.60 | 1.54 | 12.00 | 0.57 |
| **10** | 13835 | 5.66 | 197 | 1438 | 619 | 6.84 | 5.83 | 1.85 | 7.11 | 32.85 | 7.88 | 1.45 | 11.83 | 0.57 |
| **11** | 13694 | 59.81 | 826 | 1969 | 1009 | 11.72 | 17.46 | 0.61 | 8.48 | 97.46 | 4.75 | 2.14 | 21.52 | 3.10 |
| **12** | 13181 | 75.79 | 678 | 1603 | 794 | 26.10 | 15.57 | 0.53 | 7.50 | 58.94 | 3.53 | 1.93 | 17.62 | 2.24 |
| **13** | 16004 | 58.66 | 706 | 2684 | 1361 | 22.14 | 16.65 | 1.21 | 9.16 | 78.17 | 13.21 | 2.53 | 30.90 | 2.85 |
| **14** | 13821 | 62.56 | 1053 | 2492 | 1413 | 32.09 | 12.71 | 1.69 | 7.72 | 85.52 | 10.40 | 1.94 | 37.36 | 4.43 |
| **15** | 13710 | 33.72 | 667 | 2043 | 1031 | 21.32 | 11.87 | 0.98 | 8.39 | 59.61 | 9.07 | 3.69 | 23.28 | 2.90 |
| **16** | 14547 | 31.90 | 811 | 1890 | 962 | 39.18 | 12.81 | 0.99 | 8.37 | 81.24 | 8.57 | 3.58 | 24.89 | 2.54 |
| **17** | 11972 | 55.41 | 710 | 2652 | 1253 | 39.32 | 11.69 | 0.79 | 9.58 | 89.46 | 6.27 | 4.20 | 26.81 | 2.10 |
| **18** | 14044 | 45.51 | 844 | 2653 | 1329 | 21.29 | 13.53 | 0.92 | 8.74 | 62.15 | 6.27 | 3.06 | 25.97 | 3.53 |
| **19** | 15598 | 74.92 | 868 | 2047 | 1233 | 29.16 | 14.60 | 0.96 | 8.90 | 61.97 | 9.18 | 3.20 | 28.20 | 3.26 |
| **20** | 12326 | 35.84 | 594 | 1944 | 945 | 25.92 | 11.83 | 0.92 | 7.09 | 58.60 | 8.27 | 1.80 | 21.34 | 2.45 |
| **21** | 16527 | 10.85 | 916 | 2595 | 834 | 17.94 | 12.03 | 2.41 | 5.78 | 40.49 | 6.77 | 1.07 | 16.00 | 1.83 |
| **22** | 15411 | 8.55 | 390 | 2805 | 663 | 5.95 | 9.12 | 0.80 | 7.80 | 17.70 | 4.60 | 0.98 | 10.75 | 0.82 |
| **23** | 14493 | 8.03 | 491 | 2561 | 663 | 6.61 | 8.29 | 1.84 | 6.30 | 17.35 | 4.74 | 1.58 | 12.23 | 1.18 |
| **24** | 16162 | 10.04 | 636 | 3734 | 725 | 14.81 | 14.11 | 1.40 | 6.91 | 28.14 | 4.97 | 1.30 | 14.22 | 1.16 |
| **25** | 13741 | 11.52 | 414 | 1467 | 597 | 15.18 | 8.70 | 2.37 | 4.53 | 26.05 | 7.48 | 1.15 | 10.35 | 0.89 |
| **26** | 19252 | 9.72 | 625 | 2239 | 1039 | 49.17 | 12.27 | 1.38 | 5.19 | 68.89 | 10.02 | 1.35 | 16.02 | 1.81 |
| **27** | 16985 | 5.79 | 750 | 2550 | 1057 | 30.62 | 9.59 | 1.17 | 6.94 | 54.71 | 10.18 | 1.03 | 15.33 | 1.53 |
| **28** | 18294 | 7.86 | 476 | 2223 | 975 | 30.89 | 10.75 | 0.89 | 4.78 | 47.71 | 7.45 | 0.96 | 12.41 | 1.14 |
| **29** | 14868 | 5.52 | 359 | 3403 | 665 | 10.02 | 11.92 | 0.57 | 7.31 | 24.43 | 4.46 | 1.08 | 11.82 | 0.75 |
| **30** | 14862 | 6.48 | 386 | 2662 | 737 | 11.16 | 12.87 | 0.58 | 6.56 | 22.45 | 5.55 | 0.91 | 13.59 | 0.69 |
| **31** | 14516 | 4.27 | 412 | 1637 | 702 | 6.21 | 7.90 | 7.82 | 5.00 | 29.65 | 10.89 | 1.51 | 12.42 | 1.94 |
| **32** | 10826 | 3.43 | 451 | 1421 | 625 | 5.34 | 7.30 | 4.87 | 4.08 | 28.35 | 6.66 | 1.82 | 10.04 | 6.34 |
| **33** | 13393 | 5.22 | 422 | 1918 | 889 | 4.70 | 8.86 | 2.45 | 6.71 | 24.10 | 8.45 | 2.17 | 12.51 | 2.19 |
| **34** | 13044 | 4.56 | 335 | 2014 | 768 | 8.09 | 11.31 | 5.03 | 6.63 | 33.85 | 7.68 | 1.87 | 14.22 | 4.75 |
| **35** | 13334 | 4.34 | 328 | 1461 | 666 | 6.67 | 8.05 | 4.06 | 4.94 | 27.23 | 10.10 | 1.89 | 12.20 | 5.82 |
| **36** | 14850 | 3.62 | 469 | 1498 | 893 | 8.70 | 9.97 | 4.26 | 5.63 | 32.77 | 7.69 | 3.06 | 11.31 | 1.85 |
| **37** | 14386 | 3.06 | 358 | 1423 | 674 | 6.26 | 9.20 | 4.13 | 4.57 | 27.89 | 9.94 | 1.53 | 10.85 | 4.03 |
| **38** | 12925 | 3.72 | 499 | 1875 | 785 | 9.11 | 8.80 | 1.57 | 5.64 | 30.19 | 9.20 | 1.64 | 13.14 | 1.17 |
| **39** | 11690 | 2.19 | 432 | 1316 | 613 | 5.87 | 5.34 | 4.45 | 5.63 | 21.51 | 7.69 | 2.12 | 10.39 | 2.20 |
| **40** | 13738 | 3.52 | 362 | 1366 | 688 | 4.73 | 10.23 | 2.69 | 6.76 | 20.83 | 7.89 | 2.47 | 11.47 | 1.91 |

Table S2 Classification results of original data, auto-scaling and scale standard methods

| **Model** | **Preprocessing method** | **Training set** | | | | | **Test set** | | | | **Accuracy** |
| --- | --- | --- | --- | --- | --- | --- | --- | --- | --- | --- | --- |
|  |  |  | GX | GZ | HN | JX | GX | GZ | HN | JX |  |
| **LDA** | **Original data** | GX | 19 | 0 | 0 | 1 | 9 |  |  | 1 | 90 % |
|  |  | GZ | 0 | 20 | 0 | 0 | 1 | 9 |  |  |  |
|  |  | HN | 0 | 0 | 20 | 0 |  |  | 9 | 1 |  |
|  |  | JX | 0 | 0 | 0 | 14 |  | 1 |  | 9 |  |
|  | **Auto-scaling** | GX | 19 | 0 | 0 | 1 | 10 |  |  |  | 97.5 % |
|  |  | GZ | 0 | 20 | 0 | 0 |  | 10 |  |  |  |
|  |  | HN | 0 | 0 | 20 | 0 |  |  | 9 | 1 |  |
|  |  | JX | 1 | 0 | 0 | 13 |  |  |  | 10 |  |
|  | **Scale standard** | GX | 19 | 0 | 0 | 1 | 10 |  |  |  | 95 % |
|  |  | GZ | 0 | 20 | 0 | 0 | 1 | 9 |  |  |  |
|  |  | HN | 0 | 0 | 20 | 0 |  |  | 9 | 1 |  |
|  |  | JX | 1 | 0 | 0 | 13 |  |  |  | 10 |  |
| **SVM** | **Original data** | GX | 19 | 0 | 0 | 1 | 9 |  |  | 1 | 92.5% |
|  |  | GZ | 0 | 20 | 0 | 0 | 1 | 9 |  |  |  |
|  |  | HN | 0 | 0 | 20 | 0 |  |  | 9 | 1 |  |
|  |  | JX | 0 | 0 | 0 | 14 |  |  |  | 10 |  |
|  | **Auto-scaling** | GX | 19 | 0 | 0 | 1 | 10 |  |  |  | 97.5 % |
|  |  | GZ | 0 | 20 | 0 | 0 |  | 10 |  |  |  |
|  |  | HN | 0 | 0 | 20 | 0 |  |  | 9 | 1 |  |
|  |  | JX | 0 | 0 | 0 | 14 |  |  |  | 10 |  |
|  | **Scale standard** | GX | 19 | 0 | 0 | 1 | 10 |  |  |  | 95 % |
|  |  | GZ | 0 | 20 | 0 | 0 | 1 | 9 |  |  |  |
|  |  | HN | 0 | 0 | 20 | 0 |  |  | 9 | 1 |  |
|  |  | JX | 1 | 0 | 0 | 13 |  |  |  | 10 |  |
| ***k*-NN** | **Original data** | GX | 19 | 0 | 0 | 1 | 8 |  | 1 | 1 | 87.5% |
|  |  | GZ | 0 | 19 | 1 | 0 | 1 | 9 |  |  |  |
|  |  | HN | 0 | 0 | 20 | 0 |  |  | 9 | 1 |  |
|  |  | JX | 0 | 0 | 0 | 14 |  | 1 |  | 9 |  |
|  | **Auto-scaling** | GX | 19 | 0 | 0 | 1 | 10 |  |  |  | 97.5% |
|  |  | GZ | 0 | 20 | 0 | 0 |  | 10 |  |  |  |
|  |  | HN | 0 | 0 | 20 | 0 |  |  | 9 | 1 |  |
|  |  | JX | 1 | 0 | 0 | 13 |  |  |  | 10 |  |
|  | **Scale standard** | GX | 19 | 0 | 0 | 1 | 10 |  |  |  | 95% |
|  |  | GZ | 0 | 20 | 0 | 0 | 1 | 9 |  |  |  |
|  |  | HN | 0 | 0 | 20 | 0 |  |  | 9 | 1 |  |
|  |  | JX | 0 | 1 | 0 | 13 |  |  |  | 10 |  |

GX: Guangxi; GZ: Guizhou; HN: Hunan; JX: Jiangxi

Table S3 The Eigenvalues and Wilk’s lambda values for the three discriminant functions

|  | Eigenvalues | Wilk’s lambda values |
| --- | --- | --- |
| Function 1 | 31.6049 | 0.0306 |
| Function 2 | 7.8735 | 0.1126 |
| Function 3 | 1.0671 | 0.4837 |
